# Efficacy and safety of a 3D-printed arthrodesis prosthesis for reconstruction after resection of the proximal humerus: preliminary outcomes with a minimum 2-year follow-up

**DOI:** 10.1186/s12891-022-05581-6

**Published:** 2022-07-04

**Authors:** Haijie Liang, Wei Guo, Yi Yang, Dasen Li, Rongli Yang, Xiaodong Tang, Taiqiang Yan

**Affiliations:** Musculoskeletal Tumor Center, People’s Hospital, Peking University, Xizhimen Nan 11#, Xicheng District, Beijing, 100044 China

**Keywords:** Proximal humerus, Arthrodesis, Prosthesis, Tumor, 3D-printed, Axillary nerve

## Abstract

**Background:**

The purpose of this study was to investigate the feasibility of using a three-dimensional (3D)-printed arthrodesis prosthesis for reconstruction of the proximal humeral defect after tumor resection.

**Methods:**

A novel proximal humeral prosthesis was designed to restore bone continuity and shoulder arthrodesis and was fabricated via 3D printing technology. Ten patients with primary malignancies in the proximal humerus underwent intra-articular resection and replacement with this prosthesis from 2017 to 2019. Baseline and operative data, oncological and prosthetic survival, and functional status were summarized.

**Results:**

This cohort consisted of 9 males and 1 female with a mean age of 32.1 ± 16.1 years. Diagnoses included 5 cases of osteosarcoma, 3 cases of chondrosarcoma and 1 each case of undifferentiated pleomorphic sarcoma and malignant myoepithelioma. The mean operative duration, intraoperative hemorrhage and postoperative length of hospitalization were 151.5 ± 61.0 min, 410.0 ± 353.4 ml and 5.3 ± 1.9 d, respectively. The mean follow-up duration was 29.3 ± 6.4 months, with a minimum of 24 months for the surviving patients. Two patients experienced local recurrence, and four patients developed distant metastases. Detachment of the taper occurred in two patients. One was managed conservatively, and the other received amputation due to concurrent tumor recurrence. The mean MSTS-93 and ASES scores and ranges of forwards flexion and abduction were 24.9 ± 3.1, 79.4 ± 8.3, 71.3 ± 19.4°, and 61.3 ± 16.4°, respectively. The functional outcomes were independent of the preservation of the axillary nerve. Histological study of the glenoid component showed evidence of bone ingrowth at the bone-prosthesis porous interface.

**Conclusion:**

Application of the 3D-printed arthrodesis prosthesis might be a safe and efficacious method for functional reconstruction in patients who underwent resection of the proximal humerus, especially for those without preservation of the axillary nerve.

**Supplementary Information:**

The online version contains supplementary material available at 10.1186/s12891-022-05581-6.

## Background

The proximal humerus is a common site of primary and metastatic bone tumors [1]. Primary malignancies and solitary metastatic lesions require *en bloc* resection of the tumor to achieve local control. There are multiple methods for reconstruction of the proximal humerus that can be classified as prosthetic or nonprosthetic methods [1, 2]. The former includes proximal humeral prostheses [3], allograft-prosthetic composites (APCs) [4, 5], and reverse total shoulder arthroplasty (rTSA) [6–8], while the latter includes osteoarticular allografts [9, 10], arthrodesis with different kinds of autografts [11, 12], and clavicula pro humero (CPH) [13]. Although there is no consensus about the ideal method for reconstruction, proximal humeral replacement (PHR) with hemiarthroplasty is one of the most popular methods because of its convenience, good cosmetic appearance, and acceptable functional outcomes of the elbow and hand [1, 3]. However, the function of the shoulder varies greatly among patients depending on the function of the axillary nerve and muscle reconstruction [6, 14–16]. Glenohumeral arthrodesis with bone grafts can achieve permanent stability once the graft heals and might improve the motion of the arm via the movement of the scapula [12, 13, 17]. Therefore, it is a rational method for reconstruction when the axillary nerve is resected. However, the procedures of arthrodesis are complex, and complications are common, including nonunion, fracture, fixation failure and infection [1, 13].

The advent of three-dimensional (3D) printing technology has granted people more freedom to design and manufacture new prostheses with the purposes of conformational matching and osseointegration [18]. In light of the 3D-printed trabecular structure that promotes bone ingrowth, we designed a novel prosthesis that utilized the trabecular interface to achieve shoulder arthrodesis. Here, we report the preliminary results of this prosthesis. The primary objectives of this study included (1) assessment of the efficacy of the technique by evaluating the functional outcome and (2) assessment of the safety of the technique by evaluating the risks of complications. The secondary objectives included (1) assessment of implant osteointegration at the glenoid metal-bone interface and (2) assessment of the oncological outcome.

## Methods

### Data collection

This was a retrospective observational study that was approved by the institutional review board (IRB) of our hospital. Informed consent was obtained from every patient or guardian. A total of 23 surgery-naïve patients with primary malignancies in the proximal humerus underwent intra-articular *en bloc* resection and prosthetic replacement in our center between January 2017 and January 2019. In ten of them, a 3D-printed arthrodesis prosthesis was used for reconstruction. The baseline characteristics are summarized in Table 1. There were 9 males and 1 female with a mean age of 32.1 ± 16.1 years. The mean duration from symptom onset to admission was 10.8 ± 14.1 months, and the pathological diagnoses included osteosarcoma, chondrosarcoma, undifferentiated pleomorphic sarcoma and malignant myoepithelioma. Eight cases were localized, while the remaining two cases had axillary lymph node metastases on admission. Three cases were complicated by pathological fracture. The average size of the tumor was (65.2 ± 23.4) mm.

**Table 1:** Baseline and operative data of cases with primary bone malignancies in the proximal humerus

Variables	Values
Gender [N (%)]	
Male	9 (90.0)
Female	1(10.0)
Age (yr, mean ± SD)	32.1 ± 16.1
Onset duration (month, mean ± SD)	10.8 ± 14.1
Histological diagnosis [N (%)]	
Osteosarcoma	5 (50.0)
Chondrosarcoma	3 (30.0)
Undifferentiated pleomorphic sarcoma	1 (10.0)
Malignant myoepithelioma	1 (10.0)
Staging [N(%)]	
Localized	8 (80.0)
Metastatic	2 (20.0)
Pathological fracture [N(%)]	3 (30.0)
Greatest axial diameter of the tumor (mm, mean ± SD)	65.2 ± 23.4
Operative duration (min, mean ± SD)	151.5 ± 61.0
Intraoperative hemorrhage (ml, mean ± SD)	410.0 ± 353.4
Postoperative length of hospitalization (d, mean ± SD)	5.3 ± 1.9
Preservation of axillary nerve [N(%)]	4 (40.0)
Proportion of resection (%, mean ± SD)	40.3 ± 9.8

### Design of the arthrodesis prosthesis

**Fig. 1:**
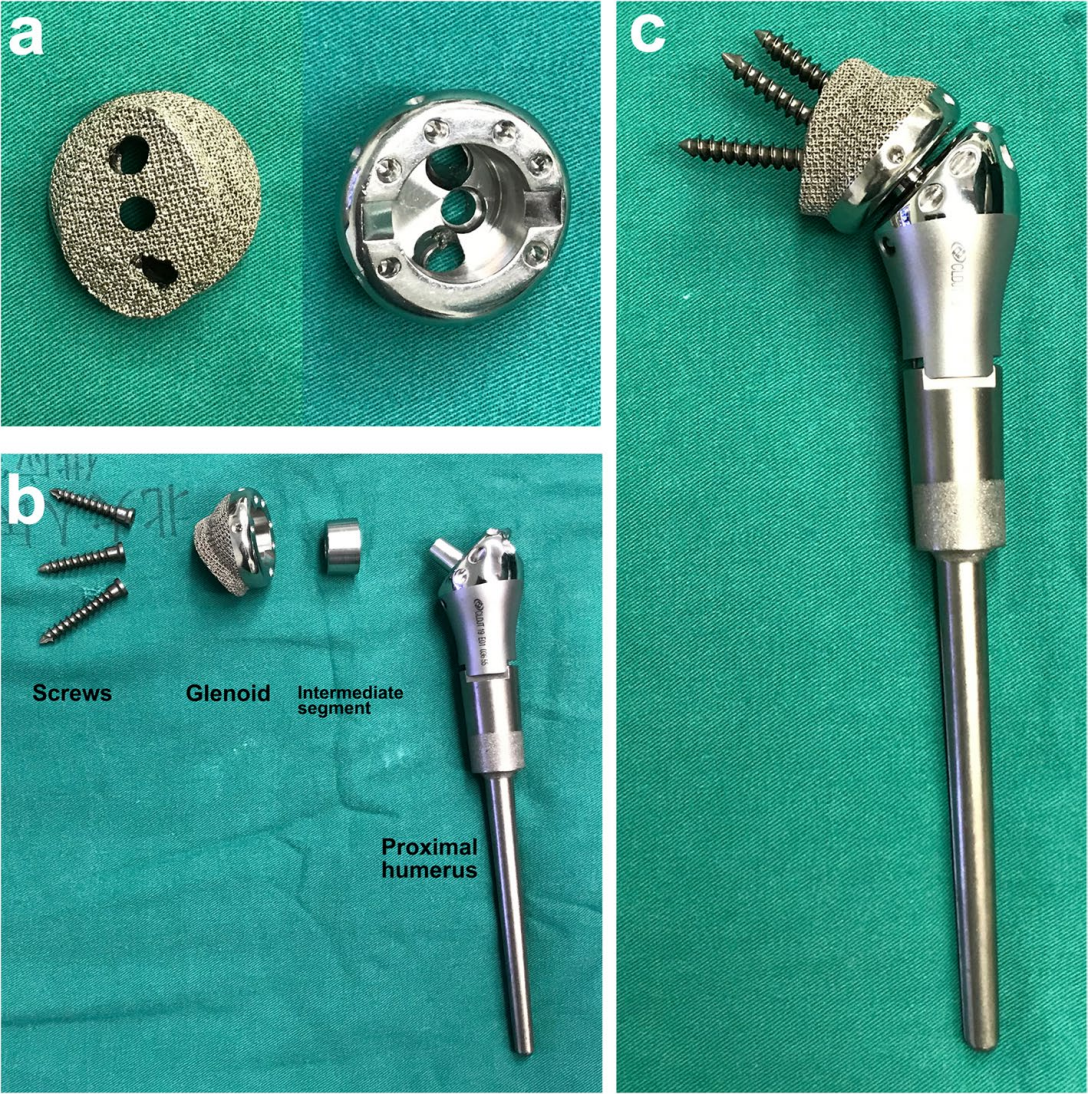
The arthrodesis prosthesis for proximal humerus. **a** Glenoid component. The contour of the outer interface (left) is designed to fit the shape of the articular surface of the scapular glenoid. The interface is of proper porosity facilitating bone ingrowth. There were three screw holes for fixation of the prosthesis to the glenoid. The inner side of the prosthesis is a Morse taper for assembly of the intermediate segment (right). Several holes around the rim of the prosthesis were used for suturing. **b** Intermediate segment and humeral component. The plug-shaped segment is used for connection between the glenoid and humeral components by the Morse taper. The humeral component is the same as the usual prosthesis for proximal humeral defects. **c** The assembled arthrodesis prosthesis

The arthrodesis prosthesis (Chunli®, Beijing, China) aimed to reconstruct the humeral defect and create a fixed glenohumeral joint. It was composed of three parts: the glenoid component, the intermediate segment, and the humeral component (Fig. 1). The new implant design was only applied to the glenoid and intermediate component, as the humeral component was off-the-shelf (Modular shoulder prosthesis, Chunli®, Beijing, China). The glenoid component was made of Ti6Al4V via the electron beam melting (EBM) technique (Fig. 1a). It was prepared in a modular manner with three consecutive sizes. The contour of the outer interface was designed to match the shape of the articular surface of the scapular glenoid. It had a proper pore size (500 μm) and porosity rate (60%) that facilitated bone ingrowth [19, 20]. There were three screw holes for fixation of the prosthesis to the scapula. The inner side of the prosthesis was a Morse taper to fix the intermediate segment. Several holes around the rim of the prosthesis were used for suturing. The intermediate segment was a metal plug with a Morse taper that connected the glenoid and humeral components (Fig. 1b). The three parts were assembled by impaction of the Morse tapers (Fig. 1c).

### Surgical procedures

**Fig. 2:**
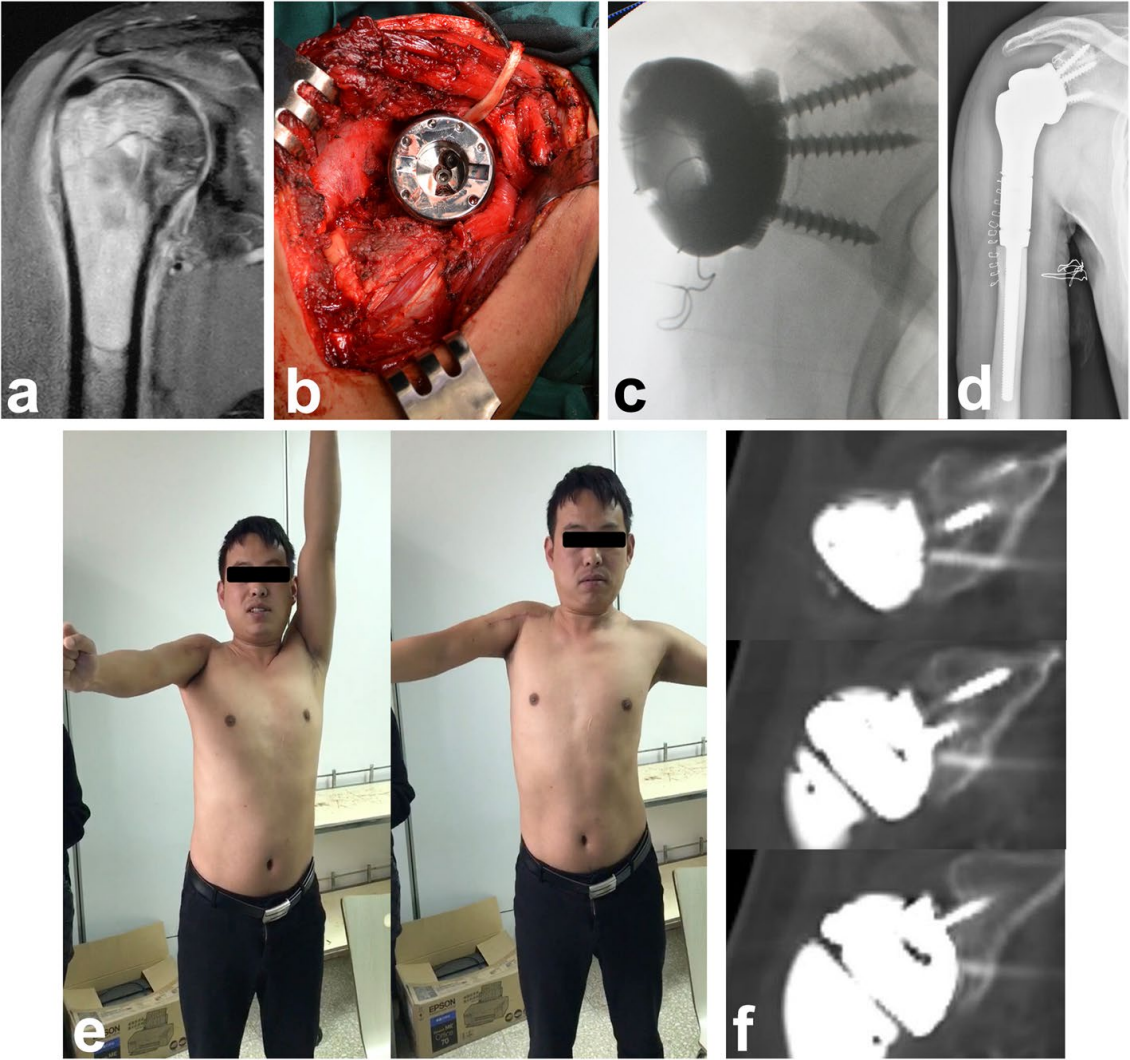
Application of the arthrodesis prosthesis. **a** A 30-year-old man diagnosed with malignant myoepithelioma in the right proximal humerus (patient #2). **b** The glenoid prosthesis was fixed to the scapula by three screws. **c** Intraoperative fluoroscopy showed good fixation of the prosthesis. **d** Postoperative X-ray showed good fixation of the prosthesis. **e** The patient showed satisfactory functional status of the right shoulder 3 months postoperatively with an MSTS-93 score of 27 and an ASES score of 81.7. **f** CT scan 12 months after surgery showed bone formation around the porous interface of the glenoid component

Resection of the proximal humerus followed the same procedures as previously reported [3, 21]. After removal of the tumor, we first dissected the joint capsule to expose the edge of the glenoid, after which we removed the articular cartilage and burnished the subchondral bone until spotty hemorrhage was seen. Then, the glenoid component was implanted (Fig. 2a, b, c). As the contour of the glenoid component matched the shape of the articular surface of the scapular glenoid very well, it was easy to locate the glenoid prosthesis. The screw trajectories were pre-determined and could be well fixed to the scapula. A tip for fixation of the glenoid component was to insert the middle screw first and then the upper and lower screws. Second, we assembled the intermediate segment and the proximal humeral component. The medullary canal of the residual humerus was prepared, and the proximal humeral component was cemented. During the period of cement polymerization, we reduced the shoulder joint, impacted the prosthesis to make the Morse tapers tight, adjusted the pronation angle of the forearm to 30° and maintained it until the cement hardened (Fig. 2d). The holes at the rims of both the glenoid component and proximal humeral component were sutured together with nonabsorbable sutures to prevent dislocation. After wrapping the prosthesis with a LARS ligament, the residual capsule and rotator cuff, as well as the insertions of the deltoid, pectoralis major and latissimus dorsi, were reattached to the LARS ligament to provide additional strength for suspension.

### Postoperative management and follow-up

Drainage tubes were removed when the volume was ≤20 mL/24 h. A third-generation cephalosporin was given during the hospital stay, which was usually 5 to 7 days, followed by oral antibiotics for 1 week. The patients wore an abduction splint for 4 weeks while exercises of the elbows and hands were allowed. Four weeks postoperatively, a sling was used, and active-assisted exercises of the shoulder were initiated. Adjuvant chemotherapy was administered 3 weeks after the operation.

Patients had regularly scheduled follow-up visits, usually every 3 months for the first 2 years, every 6 months from the third to fifth year, and yearly thereafter. Oncological status (recurrence or metastasis) and prosthetic status were assessed during each follow-up by senior orthopedic surgeons. Specifically, radiological evaluation for implant osteointegration was performed by routine CT follow-up. Implant failure was defined as removal of any part of the implant for any reason. At the latest follow-up, the patients’ survival status, ranges of motion (ROM) of the shoulder, Musculoskeletal Tumor Society (MSTS)-93 upper extremity score, and American shoulder and elbow surgeons (ASES) score were recorded (Fig. 2e) [22, 23].

### Statistical analysis

The independent t test was used for comparisons between continuous variables. Statistical analysis was performed using the Statistical Package for the Social Science (SPSS) software version 21.0 (SPSS Inc., Chicago, Illinois). A *p* value of < 0.05 was considered statistically significant.

## Results

### Operational details

All 10 patients underwent en bloc resection of the tumor with a wide margin. Four patients preserved the axillary nerve (Table 1). The mean proportion of resection was 40.3%. The mean duration of the operation was 151.5 ± 61.0 min, and the volume of intraoperative bleeding was 410.0 ± 353.4 ml. The mean postoperative length of hospitalization was 5.3 ± 1.9 d.

### Oncological survival

All of the patients were followed, and the mean duration was 29.3 ± 6.4 months (Table 2). Except for one patient who died at 16 months postoperatively, this cohort attained a minimum follow-up of 24 months. Two patients experienced local recurrence at 15 and 26 months postoperatively, with the former treated by forequater amputation and the latter by local resection. The former patient developed multiple skeletal metastases 10 months after amputation and received targeted therapy. Three other patients developed pulmonary metastases at 2, 19 and 22 months after definitive surgeries and were treated with chemotherapy. Two metastatic patients died at 16 and 31 months postoperatively, and the remaining two patients were alive with disease by the last follow-up.

**Table 2:** Follow-up data of the cases with primary bone malignancies in the proximal humerus

Variables	Values
Follow-up duration (month, mean ± SD)	29.3 ± 6.4
Survival status [N(%)]	
No evidence of disease	6 (80.0)
Alive with disease	2 (20.0)
Died of disease	2 (20.0)
Local recurrence	2 (20.0)
Time to recurrence (month, mean ± SD)	20.3 ± 7.6
Distant metastasis	4 (40.0)
Time to metastasis (month, mean ± SD)	17.0 ± 10.5
Complications [N(%)]	2 (20.0)
Soft tissue failure	0 (0)
Aseptic loosening	0 (0)
Structural failure	2 (20.0)^a^
Infection	0 (0)
Functional evaluation [N(%)]	8 (80.0)
MSTS-93 score (mean ± SD)	24.9 ± 3.1
ASES score (mean ± SD)	79.4 ± 8.3
Forward flexion (°, mean ± SD)	71.3 ± 19.4
Abduction (°, mean ± SD)	61.3 ± 16.4

^a^2 patients experienced detachment of the proximal taper of the humeral prosthesis from the intermediate segment

### Prosthetic survival

**Fig. 3:**
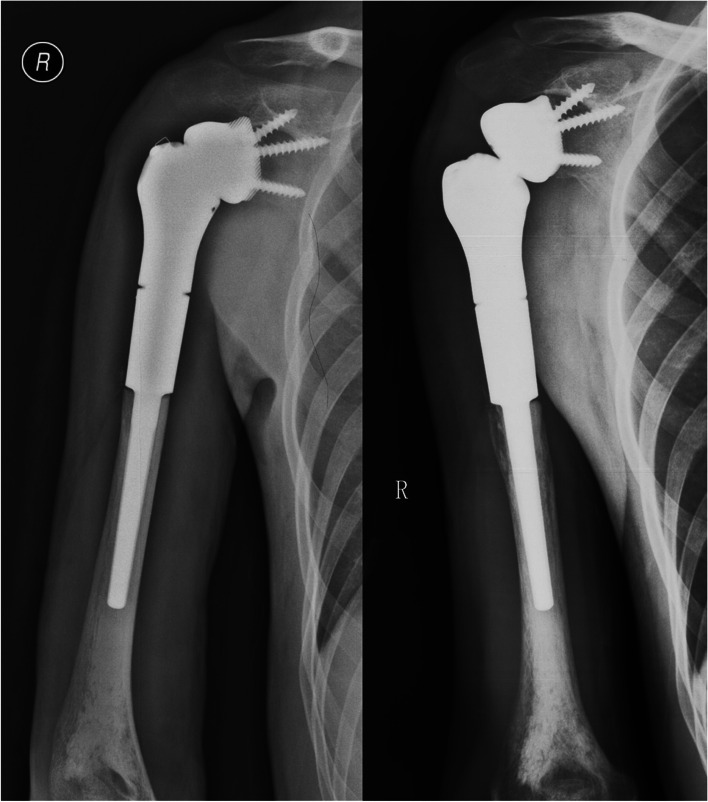
Detachment of the arthrodesis prosthesis for proximal humerus. A 20-year-old man diagnosed with osteosarcoma in the right proximal humerus underwent en bloc resection and prosthetic replacement. Postoperative X-ray showed good fixation of the prosthesis (left). Follow-up at 3 months showed detachment at the taper of the prosthesis, but the patient did not have any discomfort (right)

Two cases (20.0%) experienced detachment at the taper (Fig. 3). One patient was disease-free and refused further operations for reduction of the prosthesis, while the other patient also had tumor recurrence and was treated by forequater amputation. There were no cases of aseptic loosening, breakage, fracture, or infection in this cohort (Table 3). The rate of implant survival was 90%.

**Table 3:** Functional analysis stratified by preservation of axillary nerve

Variables	Axillary nerve preserved (*N* = 4)	Axillary nerve not preserved (*N* = 4)	*p* value
MSTS-93 score (mean ± SD)	24.8 ± 3.3	25.0 ± 3.4	0.919
ASES score (mean ± SD)	81.7 ± 11.3	77.1 ± 4.2	0.469
Forward flexion (°, mean ± SD)	76.3 ± 21.3	66.3 ± 18.9	0.509
Abduction (°, mean ± SD)	67.5 ± 19.4	55.0 ± 12.2	0.317

**Fig. 4:**
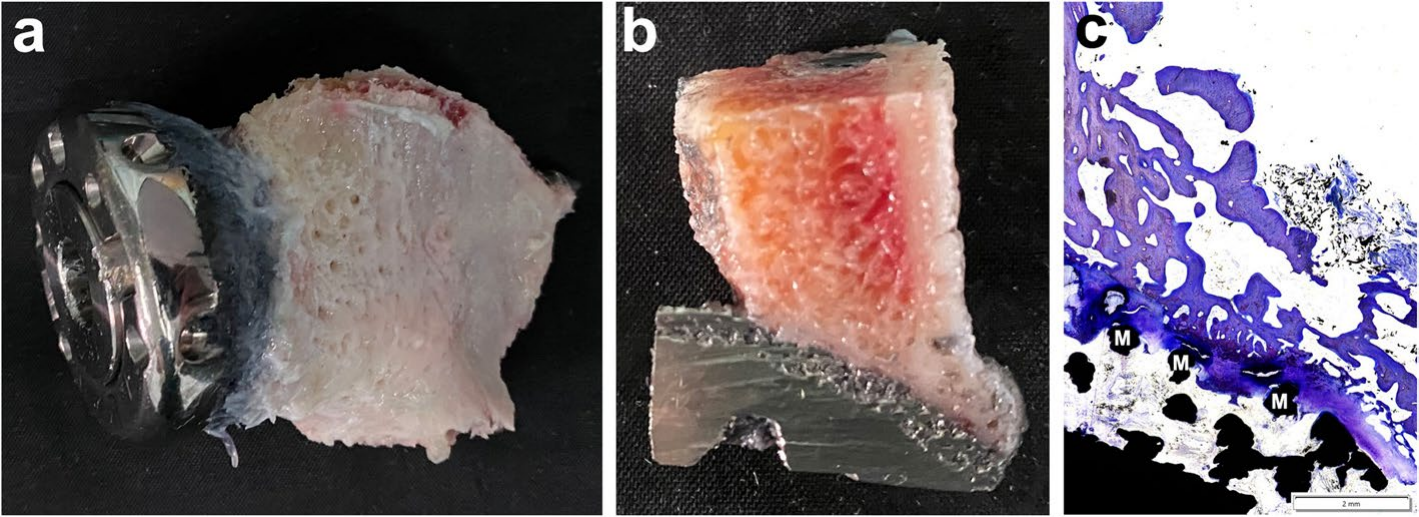
Histological study of the glenoid component of the arthrodesis prosthesis. **a** The glenoid component with part of the scapula was removed from a patient who experienced local recurrence and underwent forequarter amputation. **b** Gross view of the section of the specimen showed tight osseointegration at the bone-prosthesis interface. **c** Photomicrograph of the bone-implant interface showed new bone (stained by toluidine blue) growing into the porous morphology of the 3D-printed metallic structure. M: metallic trabeculae

In 4 of the 8 cases without complications, new bone formation around the porous interface of the glenoid component was observed via CT scan during the follow-up (Fig. 2f). Histological study of the glenoid component of the arthrodesis prosthesis was performed in patients who underwent forequarter amputation. This confirmed tight implant osseointegration at the bone-prosthesis interface (Fig. 4a, b). Photomicrographs showed new bone growing into the porous structure of the 3D-printed metallic trabeculae (Fig. 4c).

### Functional outcomes

Outcomes of functional status were obtained from 8 patients at more than 24 months postoperatively. The mean MSTS-93 score and ASES score were 24.9 ± 3.1 and 79.4 ± 8.3, respectively. The mean forward flexion and abduction angles were 71.3 ± 19.4° and 61.3 ± 16.4°, respectively. Loss of the axillary nerve did not significantly decrease the MSTS-93 score (25.0 vs. 24.8, *p* = 0.919) or the ASES score (77.1 vs. 81.7, *p* = 0.469) or the ranges of flexion (66.3° vs. 76.3°, *p* = 0.509) and abduction (55.0° vs. 67.5°, *p* = 0.317) (Table 3).

## Discussion

To improve the function of the shoulder and to simplify the arthrodesis procedures via prosthetic replacement after resection of the proximal humerus, we designed and applied a 3D-printed arthrodesis prosthesis in this study. Our preliminary results indicated that the new prosthesis could fulfil shoulder arthrodesis, achieve satisfactory functional outcomes and might be a better option for cases without preservation of the axillary nerve.

### Reflection of the design for the arthrodesis prosthesis

Prosthetic reconstruction is convenient for intraoperative manipulation, but the function of the shoulder greatly depends on the status of the deltoid muscle [24]. In contrast, arthrodesis of the joint could achieve a permanent stable shoulder with a fixed glenohumeral position, which allowed the linkage motion of the scapula-humerus composite despite the loss of axillary nerve [25]. However, the procedures of arthrodesis are complex, and mechanical failures are common afterwards (Table 4) [1, 11, 26].

**Table 4:** Comparison of the outcome of the current study to other techniques

Authors [PMID]	Reconstruction (No. of cases)	Follow-up (months)	Complications	Functional status
Grosel et al. (2019) [6] [31405716]	rTSA (10) HA (37)	Mean 27.1	HA (6 dislocation, 2 subluxation, 3 infection)	ASES score: 59 for rTSA, 63 for HA
Trovarelli et al. (2019) [27] [31389894]	rTSA (22)	Minimum 24	5 dislocation, 1 loosening	MSTS score: 29 ASES score: 81
Maclean et al. (2017) [8] [28684229]	rTSA (8)	Mean 49	1 neuropathic pain	MSTS score: 60%
Guven et al. (2016) [28] [26234664]	rTSA (10)	Mean 18.2	1 instability	MSTS score: 78.1%
Bonnevialle et al. (2015) [29] [24927883]	rTSA (8)	Mean 42	3 instability, 1 brachial plexus palsy	MSTS score: 20.25
Kaa et al. (2013) [7] [24151278]	rTSA (10)	Mean 46	3 infection, 2 loosening, 1 dislocation	MSTS score: 77%
Stavropoulos et al. (2016) [30] [27114934]	HA (19)	Mean 26.9	2 dislocation	MSTS score: 15.5
Tang et al. (2015) [3] [25604875]	HA (15) HA + mesh (14)	Mean 45	5 subluxation	MSTS score: 20 for HA, 24 for HA + mesh ASES score: 72 for HA, 85 for HA + mesh
Raiss et al. (2010) [15] [19945819]	HA (39)	Mean 38	4 dislocation, 1 shaft fracture, 2 infection, 1 loosening	MSTS score: 19
Wittig et al. (2002) [31] [11953608]	HA (23)	Median 120	8 neurapraxia, 1 loosening, 2 necrosis	MSTS score: 24–27
Barbier et al. (2017) [28699149] [13]	CPH (7)	Mean 40	5 pseudarthrosis, 2 fracture, 1 infection, 1 osteolysis, 2 frame breakage,1 nonunion, 1 ossification	MSTS score: 23
Mimata et al. (2015) [25174936] [32]	AD with VFG (5)	Mean 74.6	2 fracture	MSTS score: 71.7%
Bilgin (2012) [12] [22760395]	AD with VFG (9)	Mean 60	3 hardware prominence, 1 infection	MSTS score: 24
Hriscu et al. (2006) [33] [16894485]	AD with VFG (6)	Mean 60	2 fracture	MSTS score: 21.8
Fuchs et al. (2005) [34] [15995442]	AD (21)	Mean 132	2 wound dehiscence 1 bony prominence 2 infection 3 fracture 3 removal of screws 1 artery thrombosis 1 compartment syndrome 1 volkmann contracture	MSTS score: 23
Viehweger et al. (2005) [17] [16327688]	AD with VFG (6)	Mean 28	1 nonunion	MSTS score: 26.5
Amin et al. (2002) [11] [11953606]	AD with pedicled scapular crest graft (14)	Mean 37.3	2 neurapraxia, 3 failed fixation, 2 nonunion	MSTS score: 22.1
Current study	Arthrodesis prosthesis	Mean 29.3	2 taper detachment	MSTS score: 24.9 ASES score: 79.4

*rTSA* Reverse total shoulder arthroplasty, *HA* Hemiarthroplasty, *ASES* American Shoulder and Elbow Surgeons, *MSTS* Musculoskeletal Tumor Society, *CPH* Clavicula pro humero, *AD* Arthrodesis, *VFG* Vascularized fibular graft

The starting point of this arthrodesis prosthesis was to create a fused shoulder via a convenient operation. The immediate arthrodesis status could be achieved by assembly of the three components, while permanent arthrodesis could be fulfilled by osseointegration at the interface of the glenoid component. Radiological signs of osseointegration at the bone-implant interface were observed in half of the available cases (Fig. 2f). Histological study of the glenoid component from a recurrent chondrosarcoma case also showed new bone growing into the 3D-printed metallic structure (Fig. 4), which supported the purpose of long-term arthrodesis via osseointegration at the porous interface. The postoperative functional status of this arthrodesis prosthesis was satisfactory, with a mean MSTS-93 score of 24.9 ± 3.1 and a mean ASES score of 79.4 ± 8.3. Patients achieved an average of 71.3° forward flexion and 61.3° abduction, which justified the purpose of linkage motion by glenohumeral arthrodesis. Moreover, the functional outcomes remained stable regardless of whether the axillary nerve was preserved. Based on the results of this study, we assumed that this new arthrodesis prosthesis was rational and practical for reconstruction of the proximal humerus, especially for those without preserving the axillary nerve.

**Fig. 5:**
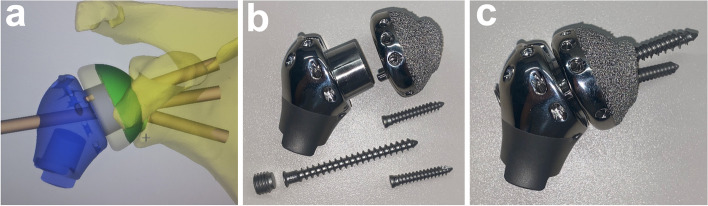
The modified arthrodesis prosthesis for proximal humerus. **a** The blueprint of the modified arthrodesis prosthesis. The humeral component was modified to have a taper that could be assembled with the glenoid component directly. Two screws were used to stabilize the glenoid component first. Then, a long screw was introduced from the humeral component, which went through the glenoid component and finally fixed to the scapula. **b** Photograph of the components of the prosthesis. Note that a nut was used to prevent withdrawal of the long screw. **c** the assembled prosthesis

However, arthrodesis prostheses did cause new forms of complications compared with PHR, i.e., detachment of the taper (Fig. 3). Detachment of the taper might be related to the weakness in resistance to torsion at the taper. A series of measures have been taken to reduce the risk of detachment. During implantation, we repeatedly examined the stability after engagement of the taper, after which we fixed the LARS ligament around the glenoid component by suturing through the holes of the rim. Meticulous suturing of the soft-tissue attachments to the LARS ligament was performed to provide additional stability. There might be a concern that the addition of LARS could increase the risk of infection. However, neither this study nor our previous report about proximal humerus replacement showed a higher infection rate when compared with historical literature [1–3]. Finally, the patients followed strict immobilization of the shoulder in an abduction brace for 4 weeks to allow scar formation around the shoulder. After taking these measures, no more cases of dislocation were observed during the follow-up in this cohort. Recently, we also improved the design of the engaging mechanism by using a long screw connecting the proximal humeral component, the glenoid component and the scapula (Fig. 5).

### Comparison between the arthrodesis prosthesis and traditional PHR

PHR with hemiarthroplasty is the most common method for segmental defects of the proximal humerus with a wide range of postoperative MSTS-93 scores [1, 2]. Teunis et al. performed a systematic review of the reconstructive techniques after proximal humerus resection, which showed that the MSTS scores of prosthetic reconstruction ranged from 61 to 77% and the implant survival ranged from 38 to 100% [2]. Another systematic review by Dubina et al. demonstrated the mean MSTS score of megaprosthesis as 72% with a reoperation rate of 10% [1]. We previously reported the outcomes of proximal humeral replacement with/without synthetic mesh augmentation in 2015 [3]. In that study, the average operative time and intraoperative blood loss were 158 min and 339 ml in the mesh group. The mean MSTS-93, ASES, ROM of flexion, and ROM of abduction were 80%, 85, 77° and 68°, respectively. In this study, the results showed that this new prosthesis did not significantly increase the operative time (151.5 min) or intraoperative hemorrhage (410 ml), indicating equivalent perioperative safety of the arthrodesis prosthesis. The mean MSTS-93 (83%) and ASES scores (79.4), as well as the ranges of flexion (71.3°) and abduction (61.3°) of this new prosthesis, were also comparable with those of our previous study and those of other studies [1–3, 21].

### Comparison between the arthrodesis prosthesis and prosthesis with reverse total shoulder arthroplasty

Most of the available prostheses with total shoulder arthroplasty for oncological cases were reverse total shoulder arthroplasty (rTSA) according to the literature [1, 6–8, 27, 28, 35]. The reported average MSTS-93 score (72.3 to 96.6%) and ROM of the shoulder (flexion: 71° to 96°, abduction: 62° to 88°) were excellent for cases of rTSA but varied across different studies [1, 8, 27, 28]. Griffiths et al. reported that in a cohort of rTSA using a Bayley Walker prosthesis, the mean MSTS score was 72.3%, and the mean ROM was 71° forward flexion and 62° abduction [35]. Grosel et al. reported that the mean range of forward flexion for rTSA patients was 85°, and the mean ASES score was 59 [6]. In a recent study of 22 patients who underwent resection of a proximal humeral tumor and replacement with a modular rTSA while preserving an innervated deltoid muscle, Trovarelli et al. reported a mean MSTS-93 score of 96.6%, a mean ASES score of 81, a mean abduction angle of 103°, and a mean forward flexion angle of 117° [27]. The results from Trovarelli et al. were much better than those of other retrospective studies [6, 7, 35], indicating the essential role of deltoid muscle in functional restoration after rTSA. Moreover, rTSA was reported to have a higher rate of complications than other arthroplasty options [1, 27, 35]. The risks of dislocation and shoulder instability could be as high as 30% in oncological cases [27, 29, 35]. Compared with rTSA, our arthrodesis prosthesis could provide better stability of the glenohumeral joint and similar upper limb function but would rely much less on the preservation of the deltoid and axillary nerve. Although this new prosthesis had a detachment rate of 20%, the overall risk of prosthetic complications was similar to that of rTSA. As a result, the arthrodesis prosthesis was an alternative choice to rTSA for cases without preservation of the axillary nerve.

### Comparison between the arthrodesis prosthesis and arthrodesis with bone grafts

Glenohumeral arthrodesis with allografts or autografts has been an alternative to prosthetic replacement in patients requiring resection of the rotator cuff, deltoid, or axillary nerve. The mean MSTS-93 scores were satisfactory, ranging from 73 to 88%, and the mean forward flexion and abduction could reach up to 80° according to the literature [1, 11, 13, 17, 33]. However, the procedures of arthrodesis are complex and tedious, and complications are common. The risks of nonunion ranged from 4.7 to 62.5% [11, 13, 17, 26, 34, 36], while the risks of infection ranged from 7 to 21% [1, 12, 34, 36]. Moreover, the risks of graft fracture ranged from 10 to 67%, and the risks of hardware failure could be up to 20% [11, 12, 32–34, 36, 37]. By applying the new arthrodesis prosthesis, we created a stable joint immediately, and permanent arthrodesis could be achieved by osseointegration at the implant-bone interface (Fig. 4). It achieved an MSTS-93 score and ROM similar to those of arthrodesis with bone grafts while avoiding the complicated procedures and risks of nonunion, infection, fracture and hardware failure, as reported in the literature [1, 11–13, 17, 26, 32–34, 36, 37].

### Limitations

There were several major limitations in this study. First, this was a retrospective study that had the intrinsic weakness of selective and recalling bias. Second, as a preliminary observation of this newly designed prosthesis, we did not establish a strict criterion for selection of the prostheses at the beginning. Third, the sample size was small, and the follow-up duration was relatively short. Although the shortest follow-up duration for the surviving patients exceeded 24 months in this study, further recruitment of patients and longer follow-up durations are needed to evaluate the risk of mechanical complications and long-term function. Finally, although there were concerns about mechanical failure because the lever arm was high with respect to upper limb weight, the osseointegration at the interface could be strong enough to endure the stress. Further biomechanical analysis is needed to justify the mechanical strength of the integrated bone-implant interface.

## Conclusion

Based on the preliminary results of this study, we concluded that the new arthrodesis prosthesis could be an alternative method for the reconstruction of bone defects after resection of a proximal humeral tumor, especially for patients without preservation of the axillary nerve.

## Supplementary Information

**Additional file 1: Supplemental Video 1.** Functional status of a patient who received replacement of the arthrodesis prosthesis at the 24-month follow-up.

**Additional file 2: Supplemental file 2.** Raw data of the eleven patients who underwent replacement of a 3D-printed arthrodesis prosthesis after resection of the proximal humerus.

## Data Availability

The datasets used and/or analysed during the current study are available from the corresponding author on reasonable request.

## References

[1] Dubina A, Shiu B, Gilotra M, Hasan SA, Lerman D, Ng VY (2017). What is the optimal reconstruction option after the resection of proximal humeral tumors? A Systematic Review. Open Orthop J.

[2] Teunis T, Nota SP, Hornicek FJ, Schwab JH, Lozano-Calderon SA (2014). Outcome after reconstruction of the proximal humerus for tumor resection: a systematic review. Clin Orthop Relat Res.

[3] Tang X, Guo W, Yang R, Tang S, Ji T (2015). Synthetic mesh improves shoulder function after intraarticular resection and prosthetic replacement of proximal humerus. Clin Orthop Relat Res.

[4] Abdeen A, Healey JH (2010). Allograft-prosthesis composite reconstruction of the proximal part of the humerus: surgical technique. J Bone Joint Surg Am.

[5] El Beaino M, Liu J, Lewis VO, Lin PP (2019). Do early results of proximal humeral allograft-prosthetic composite reconstructions persist at 5-year Followup?. Clin Orthop Relat Res.

[6] Grosel TW, Plummer DR, Everhart JS, Kirven JC, Ziegler CL, Mayerson JL, Scharschmidt TJ, Barlow JD (2019). Reverse total shoulder arthroplasty provides stability and better function than hemiarthroplasty following resection of proximal humerus tumors. J Shoulder Elb Surg.

[7] Kaa AK, Jorgensen PH, Sojbjerg JO, Johannsen HV (2013). Reverse shoulder replacement after resection of the proximal humerus for bone tumours. Bone Joint J.

[8] Maclean S, Malik SS, Evans S, Gregory J, Jeys L (2017). Reverse shoulder endoprosthesis for pathologic lesions of the proximal humerus: a minimum 3-year follow-up. J Shoulder Elb Surg.

[9] Gebhardt MC, Roth YF, Mankin HJ (1990). Osteoarticular allografts for reconstruction in the proximal part of the humerus after excision of a musculoskeletal tumor. J Bone Joint Surg Am.

[10] Getty PJ, Peabody TD (1999). Complications and functional outcomes of reconstruction with an osteoarticular allograft after intra-articular resection of the proximal aspect of the humerus. J Bone Joint Surg Am.

[11] Amin SN, Ebeid WA (2002). Shoulder reconstruction after tumor resection by pedicled scapular crest graft. Clin Orthop Relat Res.

[12] Bilgin SS (2012). Reconstruction of proximal humeral defects with shoulder arthrodesis using free vascularized fibular graft. J Bone Joint Surg Am.

[13] Barbier D, De Billy B, Gicquel P, Bourelle S, Journeau P (2017). Is the Clavicula pro Humero technique of value for reconstruction after resection of the proximal Humerus in children?. Clin Orthop Relat Res.

[14] Fujibuchi T, Matsumoto S, Shimoji T, Ae K, Tanizawa T, Gokita T, Hayakawa K (2015). New endoprosthesis suspension method with polypropylene monofilament knitted mesh after resection of bone tumors in proximal humerus. J Shoulder Elb Surg.

[15] Raiss P, Kinkel S, Sauter U, Bruckner T, Lehner B (2010). Replacement of the proximal humerus with MUTARS tumor endoprostheses. Eur J Surg Oncol.

[16] Chauhan VS, Vaish A, Vaishya R (2019). Reverse shoulder arthroplasty after failed megaprosthesis for osteosarcoma of the proximal humerus: a case report and review of literature. J Clin Orthop Trauma.

[17] Viehweger E, Gonzalez JF, Launay F, Legre R, Jouve JL, Bollini G (2005). Shoulder arthrodesis with vascularized fibular graft after tumor resection of the proximal humerus. Rev Chir Orthop Reparatrice Appar Mot.

[18] Liang H, Ji T, Zhang Y, Wang Y, Guo W (2017). Reconstruction with 3D-printed pelvic endoprostheses after resection of a pelvic tumour. Bone Joint J.

[19] Shah FA, Snis A, Matic A, Thomsen P, Palmquist A (2016). 3D printed Ti6Al4V implant surface promotes bone maturation and retains a higher density of less aged osteocytes at the bone-implant interface. Acta Biomater.

[20] Sing SL, An J, Yeong WY, Wiria FE (2016). Laser and electron-beam powder-bed additive manufacturing of metallic implants: a review on processes, materials and designs. J Orthop Res.

[21] Wei R, Guo W, Yang R, Tang X, Yang Y, Ji T (2019). Plate-prosthesis composite reconstruction after large segmental resection of proximal humeral tumors: a retrospective comparative study. Medicine (Baltimore).

[22] Enneking WF, Dunham W, Gebhardt MC, Malawar M, Pritchard DJ (1993). A system for the functional evaluation of reconstructive procedures after surgical treatment of tumors of the musculoskeletal system. Clin Orthop Relat Res.

[23] Richards RR, An KN, Bigliani LU, Friedman RJ, Gartsman GM, Gristina AG, Iannotti JP, Mow VC, Sidles JA, Zuckerman JD (1994). A standardized method for the assessment of shoulder function. J Shoulder Elb Surg.

[24] Henderson ER, Groundland JS, Pala E, Dennis JA, Wooten R, Cheong D, Windhager R, Kotz RI, Mercuri M, Funovics PT (2011). Failure mode classification for tumor endoprostheses: retrospective review of five institutions and a literature review. J Bone Joint Surg Am.

[25] van der Helm FC, Pronk GM (1994). Loading of shoulder girdle muscles in consequence of a glenohumeral arthrodesis. Clin Biomech (Bristol, Avon).

[26] Calvert GT, Wright J, Agarwal J, Jones KB, Randall RL (2015). Is claviculo pro humeri of value for limb salvage of pediatric proximal humerus sarcomas?. Clin Orthop Relat Res.

[27] Trovarelli G, Cappellari A, Angelini A, Pala E, Ruggieri P (2019). What is the survival and function of modular reverse Total shoulder prostheses in patients undergoing tumor resections in whom an innervated deltoid muscle can be preserved?. Clin Orthop Relat Res.

[28] Guven MF, Aslan L, Botanlioglu H, Kaynak G, Kesmezacar H, Babacan M (2016). Functional outcome of reverse shoulder tumor prosthesis in the treatment of proximal humerus tumors. J Shoulder Elb Surg.

[29] Bonnevialle N, Mansat P, Lebon J, Laffosse JM, Bonnevialle P (2015). Reverse shoulder arthroplasty for malignant tumors of proximal humerus. J Shoulder Elb Surg.

[30] Stavropoulos NA, Sawan H, Dandachli F, Turcotte RE (2016). Use of Ligament Advanced Reinforcement System tube in stabilization of proximal humeral endoprostheses. World J Orthop..

[31] Wittig JC, Bickels J, Kellar-Graney KL, Kim FH, Malawer MM. Osteosarcoma of the proximal humerus: long-term results with limb-sparing surgery. Clin Orthop Relat Res. 2002;(397):156-76. 10.1097/00003086-200204000-00021. 10.1097/00003086-200204000-00021 11953608

[32] Mimata Y, Nishida J, Sato K, Suzuki Y, Doita M (2015). Glenohumeral arthrodesis for malignant tumor of the shoulder girdle. J Shoulder Elb Surg.

[33] Hriscu M, Mojallal A, Breton P, Bouletreau P, Carret JP (2006). Limb salvage in proximal humerus malignant tumors: the place of free vascularized fibular graft. J Reconstr Microsurg.

[34] Fuchs B, O'Connor MI, Padgett DJ, Kaufman KR, Sim FH (2005). Arthrodesis of the shoulder after tumor resection. Clin Orthop Relat Res.

[35] Griffiths D, Gikas PD, Jowett C, Bayliss L, Aston W, Skinner J, Cannon S, Blunn G, Briggs TW, Pollock R (2011). Proximal humeral replacement using a fixed-fulcrum endoprosthesis. J Bone Joint Surg Br.

[36] Probyn LJ, Wunder JS, Bell RS, Griffin AM, Davis AM (1998). A comparison of outcome of osteoarticular allograft reconstruction and shoulder arthrodesis following resection of primary tumours of the proximal humerus. Sarcoma.

[37] Blewitt N, Pooley J (1994). Resection arthrodesis of the shoulder with autogenous fibular bone grafts. J Shoulder Elb Surg.

